# Natural Psychoplastogens As Antidepressant Agents

**DOI:** 10.3390/molecules25051172

**Published:** 2020-03-05

**Authors:** Jakub Benko, Stanislava Vranková

**Affiliations:** 1 Center of Experimental Medicine, Institute of Normal and Pathological Physiology, Slovak Academy of Sciences, 841 04 Bratislava, Slovakia; Stanislava.Vrankova@savba.sk; 2 Faculty of Medicine, Comenius University, 813 72 Bratislava, Slovakia

**Keywords:** depression, antidepressants, psychoplastogens, psychedelics, flavonoids

## Abstract

Increasing prevalence and burden of major depressive disorder presents an unavoidable problem for psychiatry. Existing antidepressants exert their effect only after several weeks of continuous treatment. In addition, their serious side effects and ineffectiveness in one-third of patients call for urgent action. Recent advances have given rise to the concept of psychoplastogens. These compounds are capable of fast structural and functional rearrangement of neural networks by targeting mechanisms previously implicated in the development of depression. Furthermore, evidence shows that they exert a potent acute and long-term positive effects, reaching beyond the treatment of psychiatric diseases. Several of them are naturally occurring compounds, such as psilocybin, *N*,*N*-dimethyltryptamine, and 7,8-dihydroxyflavone. Their pharmacology and effects in animal and human studies were discussed in this article.

## 1. Introduction

### 1.1. Depression

Depression is the most common and debilitating mental disease. Its prevalence and burden have been steadily rising in the past decades. For example, in 1990, the World Health Organization (WHO) projected that depression would increase from 4th to 2nd most frequent cause of world-wide disability by 2020 [1]. In 2008, it was ranked as the 3rd and projected to be the 1st leading cause of disability by 2030 [2].

Neurotransmitter and neuroplasticity dysregulation, the hypothalamic-pituitary-adrenal axis (HPA-axis) hyperactivation, and chronic subclinical inflammation have been identified as contributing factors along with psychological ones [2]. Selective reuptake inhibitors, targeting monoamines, have been developed to normalize most of these changes. Although these drugs have been enjoying some success and are able to induce remission, approximately one-third of patients show no response to any class, while other patients respond only partially. Because of this, new therapeutic approaches are being intensively developed. In recent years, research using deep-brain, vagus nerve and transcranial magnetic stimulation, and dissociative anesthetics or psychedelics have produced promising results [2,3,4].

Major depressive disorder (MDD) patients suffer from symptoms of depressed mood, anhedonia, sadness, lack of energy, insomnia, hypoactivity, anorexia, decreased libido, concentration, and fatigue to a varying degree throughout an episode. In atypical depression, hyperphagia and hypersomnia are present rather than their opposite [2]. Additionally, patients often ruminate excessively and manifest decreased self-confidence, guilt, and helplessness. The disorder is often accompanied by excessive anxiety or psychotic symptoms. In the most difficult cases, patients are severely disabled, unable to perform any usual activities, and neglect self-care [2]. Most of these symptoms have been found to have some neural correlates. If MDD is left undiagnosed, patients are at a greater risk of suicide, serious comorbid conditions, such as cardiovascular or neurodegenerative disease, loss of employment, or severed relationships [5,6,7].

### 1.2. Psychoplastogens

Psychoplastogens are substances capable of rapid induction of structural and functional neural plasticity with the ultimate modulation of cognitive faculties. This recently defined class [8] includes a growing number of chemically diverse compounds working through common molecular mechanisms. The concept is based upon similar effects of serotonergic psychedelics, several flavonoids, and ketamine, of which the latter has been recently approved for the treatment of resistant forms of depression [9,10]. Though they target different receptors, in the end, they promote activation of tropomyosin kinase B (TrkB) and mammalian target of rapamycin (mTOR) [8].

## 2. Pathomechanisms Relevant to the Action of Psychoplastogens

### 2.1. Monoamine Hypothesis

Monoamine hypothesis started with an accidental observation that reserpine, a drug used to treat high blood pressure, also causes behavioral depression in rodents and humans. This finding has been adopted as an experimental model of depression [11,12,13]. On a molecular level, reserpine blocks the transport of monoamines into vesicles and causes their profound depletion in the long term. This is manifested by typical symptoms of depression—anhedonia, anergia, and low mood [12,14].

The subsequent development of drugs targeting the metabolism of monoamines has resulted in the advent of tricyclic antidepressants (TCAs), monoamine oxidase inhibitors (MAOIs), selective serotonin (5-hydroxytryptamine; 5-HT) reuptake inhibitors (SSRIs), and other compounds with the combined mechanism of action. In general, these compounds lead to an increase in synaptic monoamine concentration and are able to induce complete or partial remission of the symptoms. However, the first signs of alleviation come often after several weeks of continuous treatment. Thus, there has been a need for a greater understanding of their mechanism of action [3]. Subsequently, HPA-axis dysregulation and neurogenic theory of depression have been elaborated. These help to explain the delay between the beginning of treatment and the onset of action by SSRIs. It has been shown that chronic SSRI treatment repairs these abnormalities [2,15,16].

Nevertheless, the monoamine hypothesis has withstood the testing and provides a partial explanation of the disease. Substantial evidence has been amassed during the last 50 years of research. For example, depressed patients have lower 5-hydroxyindoleacetic acid to 5-HT ratio (5-HIAA/5-HT) in cerebrospinal fluid, a sign of decreased serotonergic neurotransmission. Chronic antidepressant treatment normalizes these changes, along with symptom reduction [17]. Moreover, acute and chronic tryptophan depletion lowers 5-HT reserves, which changes reactivity to affective stimuli and leads to higher aggression or anxiety in humans [18]. Post mortem studies on suicidal patients show marked changes in the 5-HT system in the prefrontal cortex, hippocampus, or amygdala [19]. Similar results come from animal research—chronic unpredictable stress or other models of depression reduce 5-HT concentration in the prefrontal cortex and limbic system. SSRIs are able to compensate for these changes [20,21]. Furthermore, variations in genes involved in monoamine metabolism have been linked to depression [22]. Serotonin signaling is also indispensable in neurogenesis maintenance, which is another principal mechanism involved in the development of depression [16] (see below).

### 2.2. Neurotrophic Hypothesis

Impaired neurogenesis and neuroplasticity are also considered to be an important mechanism involved in the pathogenesis of depression [2]. In order to adapt behaviorally to external stimuli (including stressful ones), animals need to modify the functional connectivity of their brain networks. In the adult mammalian brain, these changes are driven by two opposite synaptic processes—long term potentiation and long-term depression, LTP and LTD, respectively. LTP is further conceptualized into two partially overlapping phases—early (e-LTP) and late (l-LTP) [23]. The former involves modification (e.g., phosphorylation, nitrosylation) of existing synaptic proteins, while the latter is characterized by expression of new synaptic proteins at both pre- and post-synaptic membranes [24,25].

The extracellular signal initiating the expression of synaptic proteins has been identified to be a brain-derived neurotrophic factor (BDNF). Its secretion is stimulated by the sole activity of neurons (both pre- and post-synaptic) expressed as calcium concentration ([Ca^2+^]). Increased [Ca^2+^] leads to activation of the cascade, including protein kinase C (PKC), mitogen-activated protein kinase (MAPK), and cAMP response-element binding protein (CREB). CREB directly up-regulates the expression of the *bdnf* gene. After its release into the synaptic cleft, BDNF binds to TrkB receptor at both pre- and post-synaptic membrane. Its stimulation leads to the activation of MAPK, mTOR, and CREB (forming a positive feedback loop) [26,27]. Activation of these pathways promotes cell survival and proliferation [25]. Besides synaptic protein synthesis, neurons treated with BDNF undergo axonal and dendritic spine sprouting and synaptogenesis. All of these changes ultimately contribute to the facilitation of synaptic communication between two neurons [25].

Chronic stress or long-term elevation of glucocorticoids are the leading etiological factors in the development of depression [2]. Their direct implication as a cause of marked atrophy of prefrontal cortex (PFC) and hippocampus is well known. On the other hand, the amygdala undergoes hypertrophy in acute depression and atrophy during long-term depression [28]. Correspondingly, chronic stress causes a decrease in BDNF concentration in these regions, along with distinctive changes in dendritic arborization and decreased cell proliferation [29,30,31,32]. Opposite effects have been found to unfold in the amygdala, also contributing to higher stress reactivity [33,34]. These changes correspond to the abnormal function of these structures, which is expressed by some symptoms of depression—higher stress reactivity, chronically elevated stress hormones, cognitive deficits, and rumination [28].

Most importantly, patients with depression have been found to have lower levels of circulating BDNF. SSRIs have been able to normalize this decrease [35,36]. Val66Met polymorphism in the *bdnf* gene leads to lower inducible expression of BDNF. Furthermore, the Met allele has been linked to increased amygdala reactivity to affective stimuli and higher trait anxiety [37]. Another study found that carriers of Met allele were more prone to anxiety and depression as a function of early life stress. Moreover, Met allele carriers had lower hippocampal and prefrontal grey matter volume, which predicted a higher risk of depression [38]. There is also some evidence that suicide cases have lower BDNF expression in the prefrontal cortex and hippocampus in comparison to controls [39] and simultaneously lower mTOR expression and proteins under its control [40]. However, there are several inconsistencies, such as differential up- or down-regulation of BDNF outside of the above-mentioned areas [41]. This might suggest that altered neurotrophic signalization is not the direct cause of depression, but rather a link in the pathomechanism.

## 3. Natural Psychoplastogens with Potential in Clinical Practice

**Figure 1:**
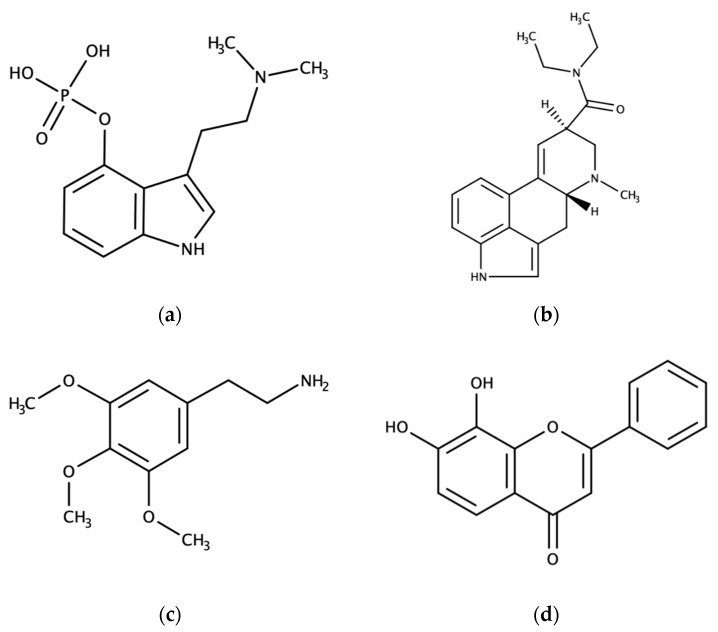
Molecular structures of: (**a**) Psilocybin; (**b**) LSD (lysergic acid diethylamide); (**c**) mescaline; (**d**) 7,8-DHF (7,8-dihydroxyflavone).

In the following section, we have summarized the molecular mechanisms and effects on animals and humans of several naturally occurring psychoplastogens, including serotonergic psychedelics and flavones (Figure 1). Serotonergic psychedelics are a broad group, based mostly around the indole ring or phenetylamine backbone. Among many others, these include psilocybin, produced by several *Psilocybe spp.*, *N*,*N*-dimethyltryptamine (DMT), produced by *Psychotria viridis*, mescaline, produced by several North American cacti, and lysergic acid diethylamide (LSD), a derivate of ergotamine, produced by *Claviceps spp.* [42]. 7,8-dihydroxyflavone (7,8-DHF) is produced by several plants, including the weed *Tridax procumbens* and a tree *Godmania aesculifolia*, commonly found in the Western Hemisphere tropics and trees in the widespread *Primula* genus [43,44]. All flavones are based around the same chemical backbone and are a subset of the larger class of flavonoids.

Research on their neuropsychiatric effects was initiated in the second half of the last century [42,45], though works on 7,8-DHF have appeared only recently [46]. All of these compounds, except psilocybin (a prodrug to psilocin), are active drugs. Furthermore, all of them show high and robust pharmacological potency without the need for modification [42]. Since psychoplastogens work through distinct mechanisms (although ultimately converging), their mechanisms and effects have been described separately.

### 3.1. Serotonergic Psychedelics

#### 3.1.1. Mechanism of Action

In general, serotonergic psychedelics possess a broad spectrum of receptor activity. Their effects are most commonly derived from the 5-hydroxytryptamine 2A receptor (5-HT_2A_R) activation [47,48]. They also jointly activate 5-HT_2B_ and 5-HT_2C_ receptors, while some of them express 5-HT_1A_R activity. Other minor effects are derived from their adrenergic, dopaminergic, and histaminergic activity [47].

5-HT_2A_R is a G_q/11_ coupled metabotropic receptor and leads to activation of phospholipase Cβ (PLCβ), hence the rise of [Ca^2+^] and activation of PKC [42]. This leads to excitatory postsynaptic currents [49] and activation of mitogen-activated protein kinase (MAPK) [50]. Interestingly, 5-HT_2A_R forms a heterodimer with metabotropic glutamate receptor 2 (mGluR2), inhibiting the activity of each other upon activation. Binding of psychedelic agonists to 5-HT_2A_R leads to a particular change in conformation and to inhibition of mGluR2, while the activation of mGluR2 by glutamate leads to inhibition of 5-HT_2A_R activity. This leads to inhibition of either phospholipase A2 (PLA2) or PLCβ, respectively [51]. This gives rise to “biased agonism” [52] and has been described to play a role in differential activation of down-stream pathways on 5-HT_2A_ receptor activation [50,53]. Though possessing similar chemical structure and binding at the same binding site, there appears to be an important difference between psychedelic and non-psychedelic 5-HT_2A_R agonists (such as lisuride or ergotamine). Serotonergic psychedelics also up-regulate PLA2, in addition to PLCβ, when compared to serotonin [50,53,54]. This is achieved by stabilization of receptor in different conformation, which preferentially interacts with G_i/o_ rather than G_q_ α-subunit of G protein heterocomplex. Psilocin has been found to activate PLA2 with 30-fold potency over PLCβ when compared to serotonin. However, the PLCβ activation is still physiologically relevant [54]. More recently, psychedelics activating 5-HT_2A_ receptors have been found to activate mTOR and increase neurite length, the number of dendrite spines, and overall network complexity [8], which is the first step, suggesting a possible therapeutic use.

#### 3.1.2. Animal Studies

5-HT_2A_Rs are typically expressed at dendrites of glutamatergic neurons in PFC, with the strongest expression in layer V pyramidal neurons. Activation of these receptors leads to an increase in excitatory post-synaptic potentials (EPSPs) [49]. Simultaneously, 5-HT_1A_R is present at the initial segment of the same neurons and drive inhibition of local currents. Functional integration of these two receptors might work as a gain control mechanism of incoming excitatory signalization, thus modulating percepts or mental representations coming to working memory [55]. Medial prefrontal cortex (mPFC) portion of these neurons projects to the basolateral amygdala, where they exert inhibitory tone via GABA neurons and decrease the amygdala reactivity [56]. These connections are thought to form a basis for fear extinction learning [57,58].

Several studies on the effects of 5-HT_2A_R agonists on anxious and depression-like behavior have been performed. Their effects are summarized in Table 1. For example, a synthetic 5-HT_2A_R agonist 2,5-dimethoxy-4-iodoamphetamine (DOI) has an anxiolytic effect in the four-plate test and elevated plus maze (EPM) [59]. Effects of natural psychoplastogens psilocybin and DMT have been explored in fear extinction protocols and forced swim test (FST). First, psilocybin has cue-potentiated fear on the first extinction trial, and DMT has shown post-acute anxiogenic effects. However, both substances have been shown to generally facilitate the extinction of fear conditioned by a cue, but not by context, which may have been mediated by its action on the amygdala [60,61]. This two-way effect is consistent with the observation that the effects of psychedelics in humans are heavily dependent on the context in which they are used. In other words, they induce emotional lability and potentiate both positive and negative emotions [62]. Both substances also decrease locomotion in EPM, suggesting that increased swimming is not due to a general increase in locomotor activity [60,61]. In addition to receptor stimulation studies, a 5-HT_2A_R knock-out mice have shown marked anxious and depressive-like behavior in comparison to wild-type mice [63]. However, one study found no effects of psilocybin and psilocin on anxiety and depressive-like behavior. Authors noted that they might have used an inappropriate model—Flinders Sensitive Line, a strain developed for the screening of antidepressant drugs. They further hypothesized that low basal expression of 5-HT_2A_R in the frontal cortex and hippocampus in this strain was potentially responsible for negative results [64]. Remarkably, these substances produce positive effects hours to days after administration, despite the fact that they are cleared out in minutes to hours. Thus, these effects must be produced by persistent changes to brain function [60].

Positive effects might be primarily exerted in mPFC, as it is responsible for fear extinction memory and direct inhibition of the amygdala [57]. Moreover, lesion studies show that the destruction of mPFC produces a depression-like phenotype in rodents [65]. Dysfunction of this region is known to play a role in depression and is related to amygdalar hyperactivation in response to negative stimuli in humans [66,67,68]. Besides its inhibitory tone on the amygdala, it also works as a hub within the default mode network (DMN), which is also found to be deregulated in depression [69,70] (see the section on human studies below).

#### 3.1.3. Human Studies

Psychedelics are well known to cause visual illusions, (pseudo-)hallucinations, synesthesia, changes in mood (both positive and negative), changes in body-space relations, alterations in time and space, depersonalization, derealization and ego dissolution [73]. These effects are completely blocked by pretreatment with selective 5-HT_2A_R antagonists (e.g., ketanserin), or atypical antipsychotics (e.g., risperidone) [74].

Several studies on therapeutic effects of psychedelics, including depression, end-of-life existential distress, substance abuse disorders, have been carried out with promising results [75,76,77]. The summary of key studies is provided in Table 2. Out of all-natural psychedelics, research on psilocybin has reached the most promising therapeutic potential. It is labeled as a “breakthrough therapy” for MDD by U.S. Food and Drug Administration and approved for phase 3 clinical trial by European Medicines Agency [45]. Additionally, phase 2 clinical trial on LSD for depression treatment is currently conducted by Bogwart and colleagues [78].

Psilocybin has produced a significant decrease in depression and trait anxiety measures in patients with moderate to severe treatment-resistant depression. The effects are sustained from 1 week to at least 6 months after treatment [76,79]. It is also used as a possible treatment of existential distress in patients with terminal cancer. In two studies, psilocybin has induced a marked decrease in depression and anxiety measures at day one after administration and lasted at least up to 26 weeks post-treatment. Moreover, overall well-being and life satisfaction are improved in most participants [77,80]. Comparable results are achieved with LSD [81,82]. Similar studies have been performed with ayahuasca, an Amazonian brew containing DMT and monoamine oxidase inhibitors. Early studies have reported on the positive psychological effects of ayahuasca in religious context [83,84]. Subsequent clinical studies have explored its effects on patients suffering from recurrent or treatment-resistant depression. Ayahuasca has produced immediate, robust, and significant decrease in anxiety and depression symptoms, at least up to 3 weeks follow-up [85,86].

These positive changes might be explained by the remodeling of brain networks involved in the development of depression. It has been found out that psychedelics cause acute disruption of the default mode network (DMN) by reduction of activity of its structures and connectivity between them [48,87]. In contrast, psilocybin promotes the post-acute strengthening of DMN [88]. Functional connectivity within DMN and activity of its constituents have been independently found to be altered in patients with depression and associated with self-centered negative rumination [69,70]. Carhart-Harris et al. [88] hypothesized that disruption of DMN by psychedelics facilitates remodeling of the connections responsible for negative rumination. Co-administration with psychotherapy helps to better integrate the insights and subsequently change patients’ attitudes towards issues [89]. Moreover, psilocybin induces positive affect in healthy volunteers, which is correlated with a decrease in amygdala reactivity to neutral and negative stimuli [90]. It also reduces the connectivity of the amygdala in emotional face discrimination [91].

### 3.2. 7,8-dihydroxyflavone and its Derivatives

#### 3.2.1. Mechanism of Action

7,8-DHF specifically binds to the TrkB receptor extracellular domain and acts as a selective TrkB agonist, mimicking the physiological actions of BDNF [43]. This is a unique mechanism of action since only a limited selection of compounds besides complex peptides can activate this receptor. Since 7,8-dihydroxyflavone is not a peptide, it is considered a small molecule activator of TrkB (only 254 Da, which is t ~ 1% of the size of the active BDNF dimer). However, these two ligands may utilize distinct mechanisms to activate TrkB receptors. Liu et al. [44] demonstrated that 7,8-DHF bound tightly to TrkB extracellular domain using two independent biophysical approaches. BDNF transiently activated TrkB, leading to receptor internalization and ubiquitination/degradation. In contrast, 7,8-DHF-triggered TrkB phosphorylation lasted for hours, and the internalized receptors were not degraded. Thus, 7,8-DHF acted as a robust TrkB agonist, passing the blood-brain barrier and exhibiting promising therapeutic efficacy in a variety of neurological and psychological diseases. A structure-activity relationship study has shown that the catechol moiety in 7,8-DHF is critical for its agonistic effect [93]. Liu et al. [46] showed that the catechol group was extensively conjugated by glucuronidation, sulfation, and methylation in the liver, leading to poor oral bioavailability [46]. Chen et al. [72] synthesized 7,8-DHF derivatives to modify the catechol group. They found that prodrug R13 exhibited good absorption and was readily hydrolyzed into 7,8-DHF in liver microsomes. Notably, R13 had a very long half-life, and it improved 7,8-DHF’s oral bioavailability from 4.6% to ∼10.5%. The plasma concentration and brain exposure were also significantly enhanced. Chronic oral administration of R13 alleviated Aβ deposition, attenuated the loss of hippocampal synapse, and ameliorated memory deficits in a dose-dependent manner.

Although 7,8-DHF has been able to promote TrkB activation in vivo, it has failed to do so in vitro [94]. The authors reported the absence of TrkB, extracellular signal-regulated kinases (ERK), and AKT phosphorylation in embryonic cortical neuron cultures. They proposed that discrepancies might have been caused by the conversion of 7,8-DHF to some other active metabolite, transactivation of TrkB through activation of G proteins or receptor tyrosine kinases. Furthermore, they proposed that TrkB phosphorylation by 7,8-DHF in vivo might be a methodological artifact [94].

#### 3.2.2. Animal Studies

It has been shown that systemic administration of 7,8-DHF can induce BDNF-like behavioral phenotypes, such as enhanced learning and memory and antistress or antidepressant-like effects in rodents in a TrkB-dependent manner [71,93,95]. Key studies are summarized in Table 1. Administration of 7,8-DHF into BDNF conditional knockout mice has also triggered TrkB activation in the BDNF-depleted cortex. Hence, 7,8-DHF induces TrkB activation in the mouse brain in a BDNF-independent manner [43]. Intraperitoneal injection of 7,8-DHF improved behavioral tests, specifically, forced swim test and tail suspension test, in the mouse model of depression [96]. Since not many studies on the effects of 7,8-DHF in depression models were performed, it is worthwhile to mention other studies which explore its application in models of diseases with neurodegenerative component (as depression partially is). For instance, 7,8-DHF prevented the degeneration of the vestibular ganglion in mice lacking BDNF and promotes axon regeneration [97]. It also rescued synaptic plasticity in cognitively impaired aged rats [98]. Remarkably, 7,8-DHF has regulated the BDNF deficiency-induced learning and memory phenotypes in mice genetically deficient for the proprotein convertase PC7 [99]. Mounting evidence has also demonstrated neuronal survival in various models, including dopaminergic neurons [43], retinal ganglion cells [100], and motoneurons [101], after 7,8-DHF administration. 7,8-DHF also has potent neurotrophic effects in various neurological disease models, including stroke [102], and neurodegenerative diseases, including Huntington’s disease [103] and Alzheimer’s disease (AD). Several studies have indicated that 7,8-DHF rescues memory deficits in different AD animal models, restoring deficient TrkB signaling without affecting endogenous BDNF levels. It has improved spatial memory and increased thin spine density in an AD mouse model [104,105]. These findings provide a groundwork for proceeding the prodrug into clinical trials.

#### 3.2.3. Human Studies

To date, no human studies on the treatment of MDD with 7,8-DHF have been performed. Since the compound has shown promising results in animal models, it is reasonable to expect some amelioration (at least in the cognitive domain) in these patients too.

## 4. Safety

Physiological side effects caused by psychedelics are usually moderate at best. Most commonly, they include clinically insignificant elevation of heart rate and blood pressure, headaches/migraines, GIT discomfort with nausea, vomiting, or diarrhea (especially in the case of ayahuasca) [48,77,80,85,86]. Their toxicity is particularly low [106].

Psychological adverse effects are of greater interest. Psychedelics may cause transient but tolerable confusion, perceptual pseudo-/hallucinations, vivid imagination, dysphoria, anxiety, fear, and paranoia [48,74,77,80,85,86]. For example, Ross et al. [77] commented that at the time of their study, approximately 2000 doses of psilocybin had been safely administered in the US and Europe without any serious adverse event. It is important to note that in these studies, the administration was performed under carefully controlled conditions with strict exclusion criteria and with medical and psychological aid at hand. In case of severe adverse events, the staff is usually equipped with tranquilizers (benzodiazepines and antipsychotics). Nevertheless, population studies indicate that there is no association between life-time psychedelics use and any mental illness, suicidal ideation, or suicide attempts [107]. Even more surprisingly, the use of psychedelics has predicted a lower need for mental health treatment in the past year in comparison to the general population [107].

Despite their relative safety, study protocols should implement strict exclusion criteria for susceptible individuals with a personal or family history of schizophrenia, mania, or bipolar disorder (BD) [106]. Susceptible individuals (especially BD patients in the first depressive episode) without personal or family history might be at risk of developing of (at least) transient psychosis.

In general, authors conclude that psychedelics are generally safe [48,77,80,85,86]. No prolonged psychotic-like reaction has been reported in any trial in the last 30 years [108]. On the other hand, studies on these substances from the pre-prohibition era show that the condition of people with existing psychotic diseases (schizophrenia and mania) aggravates after treatment [106].

## 5. Conclusions

The development of novel therapeutic compounds for the treatment of depression has trickled down in the last decade. Since pharmaceutical companies are reluctant to engage in high-risk development of drugs for psychiatric illnesses, it is of utmost importance to look for novel therapies [109]. The recent adoption of ketamine for treatment-resistant depression is an important improvement of the situation. Growing evidence on the effects of serotonergic psychedelics suggests that they might prove to be an important psychiatric medication of the future. Similar promising results from studies on methylenedioxymethamphetamine (MDMA), currently in phase 3 of clinical trials, confirm and extend the validity of fast-acting antidepressants [45,110]. Furthermore, the risk of adverse effects and substance abuse of these compounds is relatively low and/or short-lasting. Nevertheless, such concerns need to be taken into account [89,111]. It is tempting to consider that 7,8-DHF is a better alternative to serotonergic psychedelics because studies have not reported on typical proxies of hallucinogenic properties in animals. These proxies include head-twitch response, prepulse inhibition disruption (PPI), or drug discrimination essays. Only one relevant study has shown that 7,8-DHF suppresses PPI deficits induced by methamphetamine [112]. This advantage is speculative at best. On the other hand, BDNF-TrkB signaling appears to be involved in neuropathic pain and promotes the strengthening of pathological nociceptive signals at the level of the spinal cord and anterior cingular cortex (ACC) after nerve injury. This might be a contraindicated condition if 7,8-DHF is ever used in clinical studies [113,114].

A more viable alternative to classical psychedelics and 7,8-DHF might have been found by Olson lab [115]. They synthesized DMT isosteres with psychoplastogenic activity comparable to ketamine but with lower hallucinogenic potential [115]. More studies are needed to confirm its effects and assess their safety. But still, mind-altering properties of psychedelics might be essential since they mediate therapeutic effects [75,80].

Compared to conventional, long-term treatment with reuptake inhibitors, these substances produce therapeutic effects after two to three therapeutic sessions, carry a low risk of non-compliance, and produce no withdrawal symptoms.

## Figures and Tables

**Table 1:** Key animal studies on behavioral effects of psychoplastogens.

Study	Substance	Dosage	Timing	Strain	Main Findings
Andero et al., 2011 [71]	7,8-DHF	5 mg/kg i.p.	60 min before procedures	C57BL/6J mice	Activation of amygdalar TrkB receptors by 7,8-DHF. 7,8-DHF enhanced fear acquisition and extinction.
Cameron et al., 2018 [60]	DMT	10 mg/kg i.p.	60 min before procedures; in FST, 30 min after pre-test and 6 h and 1 h prior to test	SD rats	Decreased exploratory behavior of rats in OF and EPM. The initial increase in freezing in fear conditioning was affected, but contextual and cued fear memory was not affected in the following days. Decreased freezing in cued fear extinction but not in context fear extinction. Reduced immobility in FST was equal to the effects of ketamine.
Catlow et al., 2013 [61]	psilocybin	0.1–1.5 mg/kg i.p.	1 d before procedures	C57BL/6J mice	Acquisition of conditioned fear response was intact in C57BL/6J mice. In extinction learning, low doses (0.1 mg/kg and 0.5 mg/kg) showed initial increase in freezing, but in later trials, they facilitated fear extinction. Low dose (0.1 mg/kg) caused trend towards increase, but high dose (1 mg/kg) caused decrease in hippocampal neurogenesis.
Chen et al., 2018 [72]	R13 - 7,8-DHF prodrug	7.25–24.6 mg/kg p.o.	daily, from 2 to 5 mo of age	5XFAD mice	Increased bioavailability, a reversal of hippocampal synaptic loss, an increase in hippocampal LTP magnitude, a decrease in Aβ deposition in the hippocampus and frontal cortex, and improvement in spatial learning and memory in 5XFAD mice.
Dhonnchadha et al., 2002 [59]	DOI	0.25–4 mg/kg i.p.	30 min before procedures	Swiss mice	Anxiolytic effect in four-plate test (increase in punished passages) and EPM (increased number of open arm entries). No effects in light/dark box test.
Jefsen et al., 2019 [64]	psilocin, psilocybin	0.5–2 mg/kg for psilocin; 2–10 mg/kg for psilocybin	single dose 4–24 h before procedures; 3 doses in 3 d, last injection 8 d before procedures	FSL rats	Psilocin and psilocybin failed to promote any effects on behavior in FST.

7,8-DHF (7,8-dihydroxyflavone); TrkB (tropomyosin kinase B); DMT (*N,N*-dimethyltryptamine); FST (forced swim test); OF (open field); LTP (long term potentiation); Aβ (amyloid β); DOI (2,5-dimethoxy-4-iodoamphetamine); FSL rats (flinders sensitive line rats).

**Table 2:** Key human studies on the clinical effects of psychoplastogens.

Study	Substance	Dosage	Timing	Design	N	Main Findings
Carhart-Harris et al., 2016, 2017, 2018 [76,79,88]	psilocybin	10 and 25 mg p.o.	low and high dose 7 days apart at the beginning of the sessions	open-label; no control group; low dose for safety assessment	12–20	The decrease in depressive symptoms and anxiety at 1 week, 3 months, and 6 months post-treatment. Decreased CBF in structures of the temporal lobe, including the amygdala, 1-day post-treatment. The decrease in amygdala CBF correlated with a reduction in depressive symptoms.
Davis et al., 2020 [92]	psilocybin mushrooms, psilocybin, LSD, ayahuasca, mescaline, DMT, etc.	single recreational dose	questionnaires 3 mo before and 3 mo after the psychedelic experience	an internet-based cross-sectional survey of recreational users	985	Acute effects were associated with decreases in depression and/or anxiety. The decrease was fully mediated by psychological flexibility.
Gasser et al., 2014, 2015 [81,82]	LSD	20 µg (active placebo) or 200 µg	single dose at the beginning of the session	open-label; cross-over; with initial blinding	12	Decrease in state anxiety at 2 months and sustained at 12 months follow-up. No changes in trait anxiety. Subjective reports of a higher quality of life.
Griffiths et al., 2016 [80]	psilocybin	1 or 3 mg/70 kg (active placebo) and 22 or 30 mg/70 kg p.o.	low or high dose at the beginning of 2 sessions; 38 d between the sessions	randomized, double-blind, cross-over trial	51	High dose produced a decrease in depressive symptoms and anxiety in cancer patients with life-threatening diagnoses. It also increased the quality of life, life meaning, and optimism. Changes were sustained at a 6-month follow-up. The effects were mediated by mystical-type experiences on session day.
Palhano-Fontes et al., 2019 [86]	DMT, harmine, harmaline, tetrahydroharmine (ayahuasca)	0.36, 1.86, 0.24, 1.2 mg/kg p.o.	single dose at the beginning of the session	randomized, double-blind, cross-over trial	29	Substantial decrease in depression symptoms on day 1, day 2, and day 7 post-treatment. Response rate was 64% vs. 27% in favor of ayahuasca. Remission rate showed a trend towards significance at day 7 (36% vs. 7%).
Ross et al., 2016 [77]	psilocybin	0.3 mg/kg p.o.	single dose at the beginning of the session	randomized, double-blind, cross-over trial	29	Immediate, substantial, and sustained decrease in depression symptoms and anxiety in cancer patients. The effects were significant after session 1 until cross-over 7 weeks later. After cross-over, both groups showed a substantial reduction in depression symptoms and anxiety compared to baseline. These effects were sustained at a 6.5-month follow-up and were mediated by mystical-like experiences on session day.

CBF (cerebral blood flow); LSD (lysergic acid diethylamide); DMT (*N*,*N*-dimethyltryptamine).
